# Study on laser powder bed fusion of nickel-base alloy of G-surface structure: scanning strategy, properties and compression properties

**DOI:** 10.1038/s41598-021-86213-2

**Published:** 2021-03-25

**Authors:** Bo Qian, Hongri Fan, Jianrui Zhang, Tengfei Li, Jiangtao Xi, Zhijun Qiu

**Affiliations:** 1 School of Mechanical and Automotive Engineering, Shanghai University of Engineering Science, Shanghai, 201620 China; 2 School of Mechanical and Power Engineering, East China University of Science and Technology, Shanghai, 200237 China; 3 School of Mechanical, Materials, Mechatronic and Biomedical Engineering, University of Wollongong, Wollongong, Australia

**Keywords:** Materials for devices, Materials for energy and catalysis, Techniques and instrumentation, Theory and computation, Materials science, Nanoscale materials, Nanotoxicology, Techniques and instrumentation, Mechanical engineering

## Abstract

Aiming at laser powder bed fusion of GH3536 nickel base alloy, the effects of different scanning strategies on microstructure, porosity and mechanical properties were explored. In the aspect of microstructure and micro hardness of the sample, three scanning strategies had little difference; in the aspect of macro mechanical properties of the sample, the slope subarea scanning was better than the helix and island scanning. On this basis, the slope subarea scanning was selected as the optimal scanning strategy to form the G-surface structure, and the compression performance of G-surface was studied. The results showed that: (1) the compression performance of G-surface structure was smaller than that of solid structure, The compression strength of G-surface can only reach about 20% of solid structure: the average strength value of G-surface is 220 MPa, solid structure is 1.1 GMpa; while G-surface structure had a smooth compression curve, which indicated the good energy absorption characteristics; (2) with the increase of wall thickness, the mechanical performance of G-surface structure was also enhanced, while the energy absorption capacity was constantly reduced; (3) with the same wall thickness, the compression performance of sample in building direction (BD) is higher than that in horizontal direction (HD).

## Introduction

Laser powder bed fusion (LPBF) is a kind of additive manufacturing technology. The complexity of LPBF processing technology, such as the combination of rapid solidification and thermal cycle, leads to the better mechanical properties of manufactured parts by LPBF^1^. Due to the advantages in the manufacture of complex geometric parts and small batch production. LPBF has great potential to replace the traditional subtractive manufacturing technology, especially the application of GH3536 alloy in high temperature. GH3536 is a high ferric nickel base superalloy reinforced by the solid solution of chromium and molybdenum. It has excellent corrosion resistance and oxidation resistance, good creep strength below 900 ℃, good cold (hot) processing formability and welding performance^2–4^. Since GH3536 is often used in high temperature, high stress and cyclic loading conditions, It is very important to control the cracks development and porosity formation of the microstructure of materials^5–12^. In this paper, the process of GH3536 nickel base alloy was studied by the original designed and manufactured LPBF equipment, and the influence of different scanning strategies on the microstructure, porosity and performance of GH3536 nickel base alloy was explored. In the self-designed LPBF equipment, the best scanning strategy was used to verify the formability of Gyroid minimal surface; and the compression performance of Gyroid minimal surface was explored^13–16^. G-surface, namely minimal surface, can be defined by two mathematical descriptions of the area description and the curvature description. For the area description, G-surface is defined as the surface with the smallest area satisfying all external constraints (such as surface circumference or external stress conditions). For the curvature description, G-surface is defined as the surface with the average curvature of zero. The definition of average curvature is as follows: the average curvature is the average of the main curvature which is the maximum and minimum curvature of a point in space on any surface. Triply periodic minimal surface is the periodic minimal surface function, which show periodic changes along the X, Y, Z axes in Euclidean space. With the help of periodicity of triply periodic minimal surface, smooth and fully connected porous structures can be formed^17^.

## Experiments and materials

### Experimental equipment

**Figure 1:**
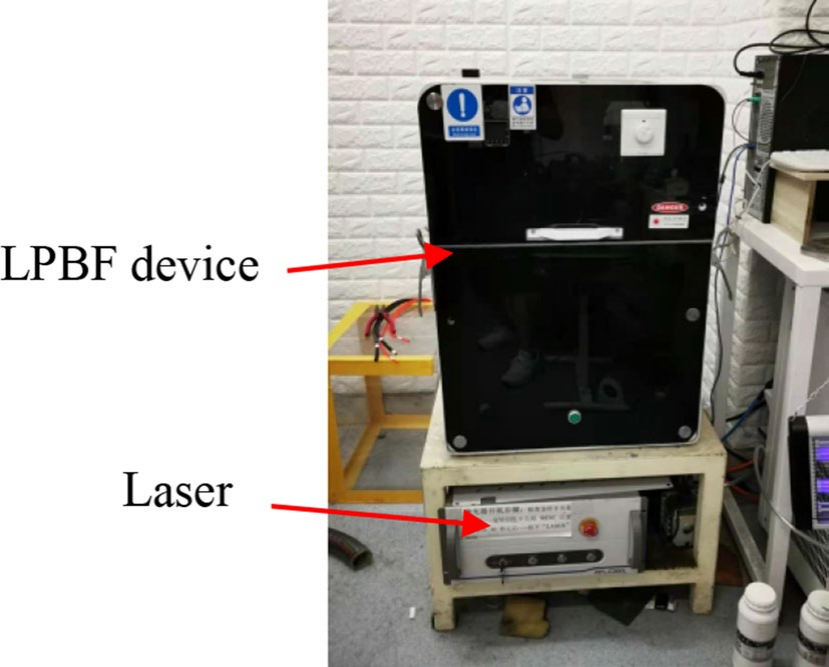
LPBF equipment.

The self-developed laser powder bed fusion additive manufacturing prototype was selected as the experimental device, and the fiber laser was used. The maximum laser power can reach 250 W, the laser wavelength was 1080 nm, and the minimum laser spot diameter was 70 μM. In the manufacturing process, nitrogen was used as the protective gas to isolate oxygen and prevent the oxidation of weld pool structure during the LPBF laser high temperature processing. Figure 1 shows the equipment structure.

### Experimental materials

**Figure 2:**
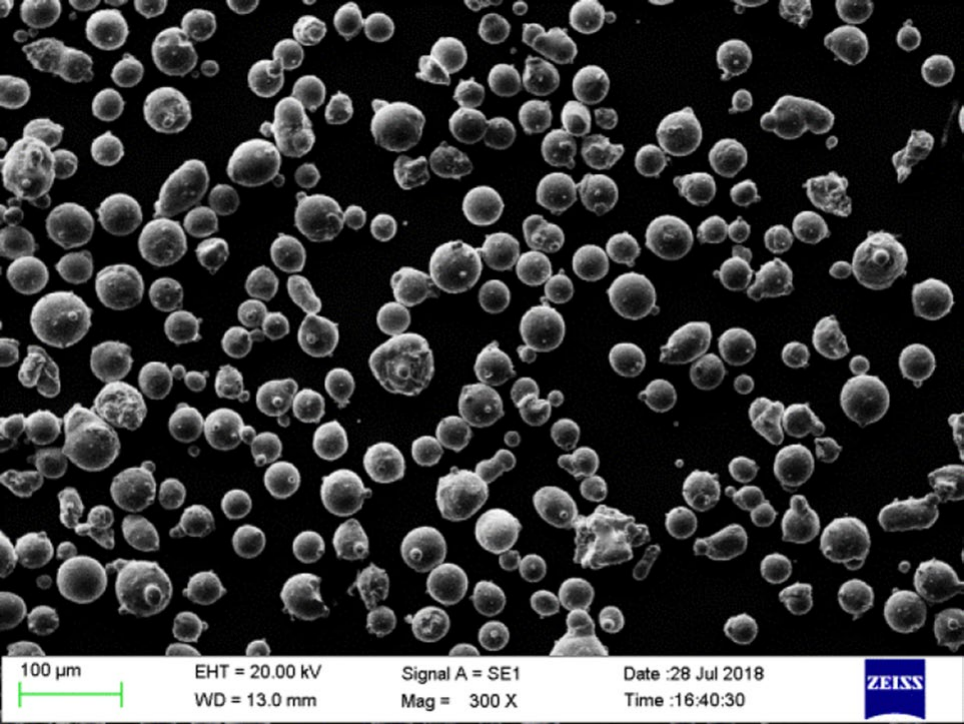
SEM image of powder.

**Figure 3:**
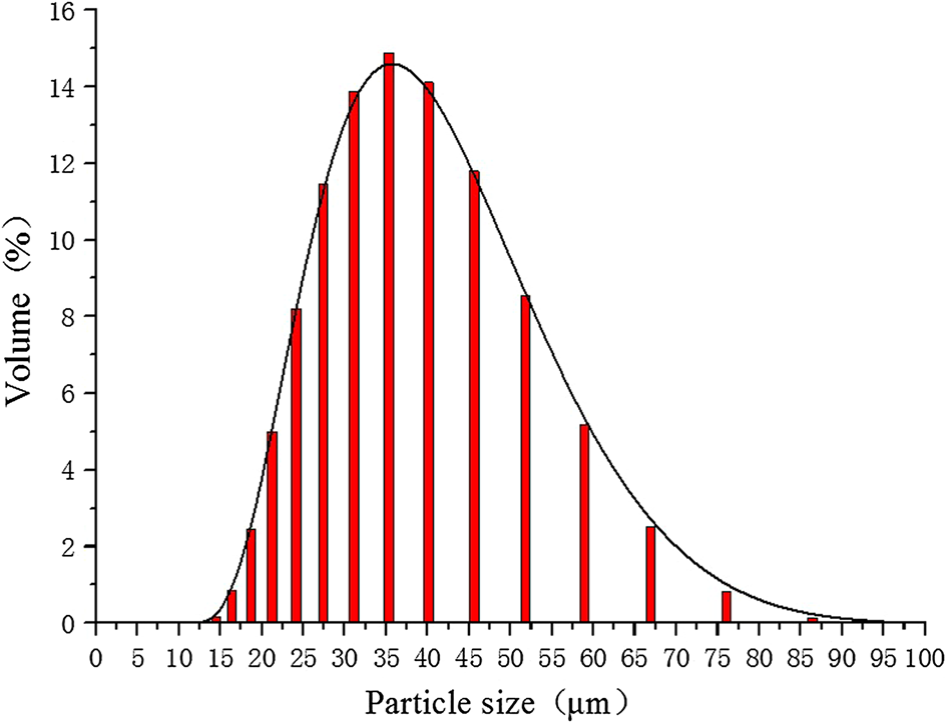
Particle size distribution of powder.

GH3536 nickel base alloy was used as the metal powder in the experiment^11^. As shown in Fig. 2, its particle size range was 18–64 μm, particle size distribution was D10 = 24.7 μm, D50 = 37.8 μm, D90 = 57.7 μM. Figure 3 shows the particle size distribution. GH3536 nickel base alloy powder contains various elements, including Ni, Cr, Fe, Mo, Co, W, C, Si and Mn. Table 1 shows the specific element content of GH3536 nickel base alloy powder.

**Table 1:** Chemical composition of the GH3536 powder.

Element	Cr	Fe	Mo	Co	W	C	Mn	Si	Ni
Mass fraction/wt%	22	18	9	1.8	0.9	0.1	Max1	Max1	Bal

### Process parameters

**Figure 4:**
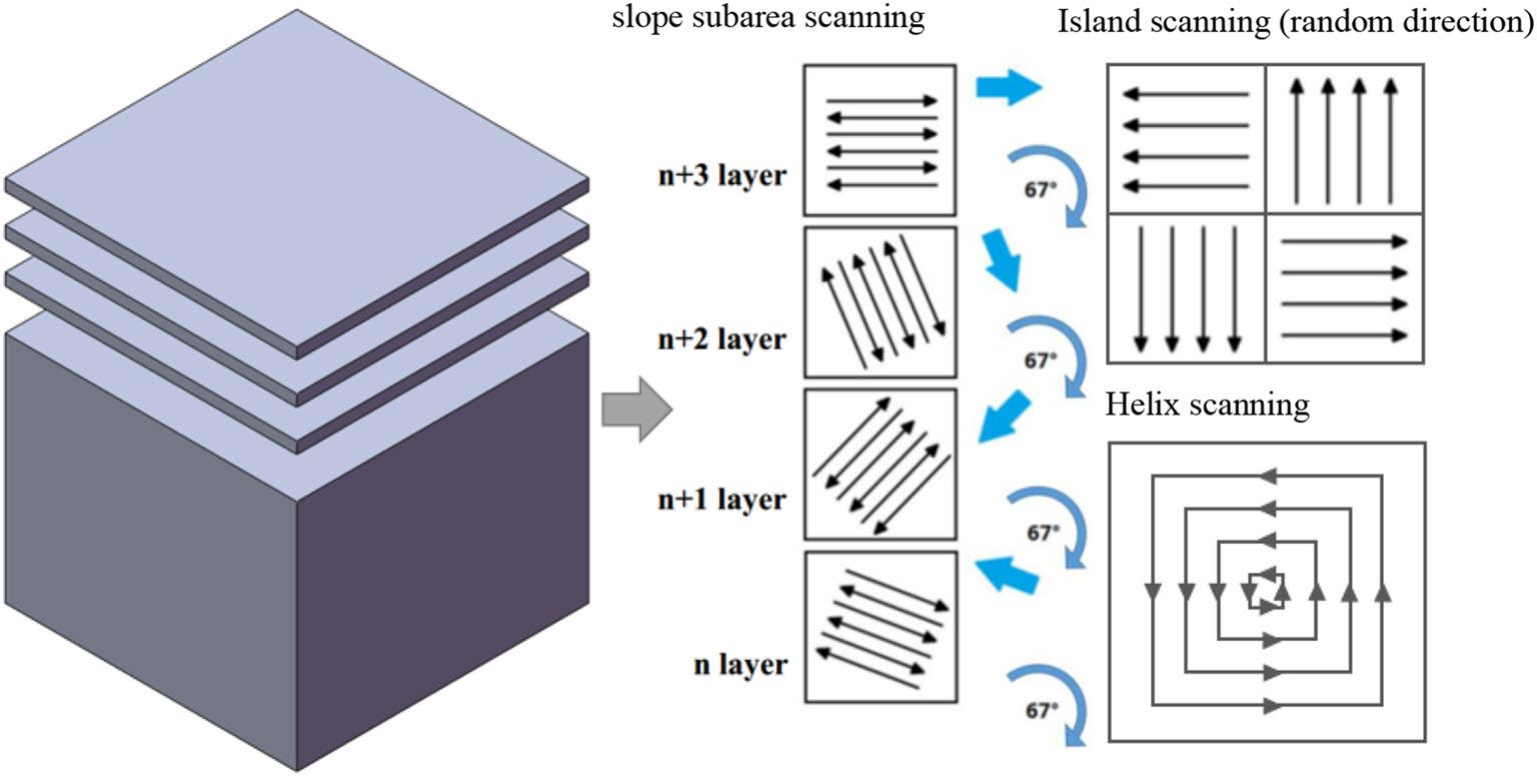
Schematic diagram of different scanning strategies.

In this experiment, three different scanning strategies, namely slope subarea scanning, island scanning and helix scanning, were used to explore the influence on the microstructure and properties of the samples. Figure 4 illustrates the schematic diagram of different scanning strategies. Except for different scanning strategies, other process parameters were the same in this experiment. Table 2 shows other process parameters.

**Table 2:** Experimental process parameters.

Process parameters	Value
Laser power *P*	200 W
Scanning speed *v*	1000 mm/s
Scanning distance *m*	0.06 mm
Thickness of powder layer *H*	0.035 mm

### Experimental model

The samples for experimental model were mainly divided into block samples, tensile samples and compression samples.

**Figure 5:**
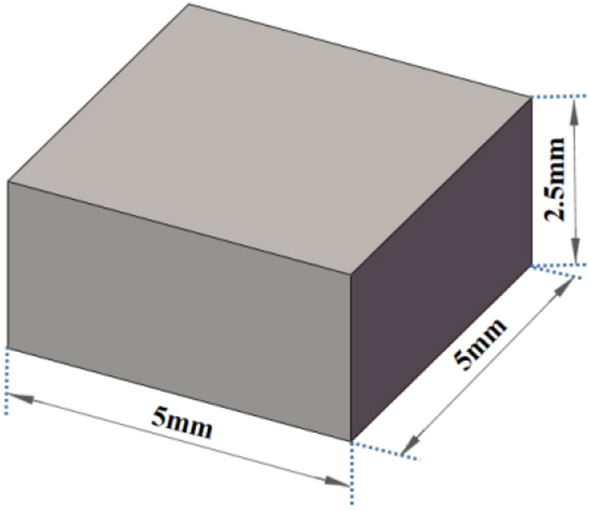
Size of metallographic sample model.

**Figure 6:**
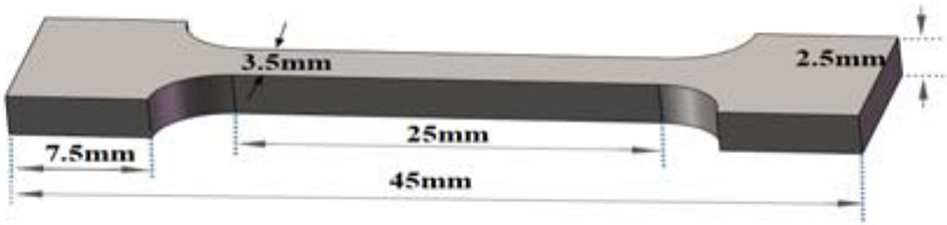
Size of tensile sample model.

**Figure 7:**
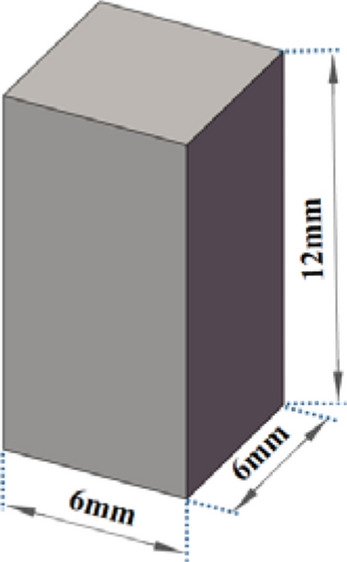
Model size of solid compression sample.

1. Block sample model:
  The block sample model was selected to print a simple square block with the size of 5 × 5 × 2.5 mm, as shown in Fig. 5. Then micro-structure and organizational structure of the sample were observed by the metallographic microscope and electron microscope.
2. Tensile sample model:
  Figure 6 shows the dimension of the tensile sample model. Instron universal material testing machine was used to measure tensile performance of the sample, and the corresponding tensile performance curve and data were obtained.
3. Compression sample model:
  Solid compression model:
  Figure 7 shows the model size of the solid compression sample. The standard compression size of 6 × 6 × 12 mm was selected. The compression performance of the sample was tested by Instron universal material testing machine, and the corresponding compression performance curve and data were obtained.
4. Compression model with the complex surface:
  G-surface structure was selected as the compression sample model with the complex surface^17,18^. As a branch of average curvature surface, G-surface is a kind of mathematical surface with 3-D periodicity, zero average curvature and large surface area. G-surface divides the space into two interwoven subdomains while keeping the cavity open. The mathematical implicit function relation of a minimal surface is described as follows:
  1 $$ \Phi_{G} \left( {x,y,z} \right) = \cos \left( {\omega x} \right)\sin \left( {\omega y} \right) + \cos \left( {\omega y} \right)\sin \left( {\omega z} \right) + \cos \left( {\omega z} \right)\sin \left( {\omega x} \right) = c $$
  where c is a constant which controls the shape of the surface and the surface area of the structure. If c = 0 , it is the G surface. *ω* is a coefficient, which indicates the number of periodicity. In other words, if *ω* = 2π, there is a minimal surface element in a 1 × 1 × 1 cube with a period of 1.

**Figure 8:**
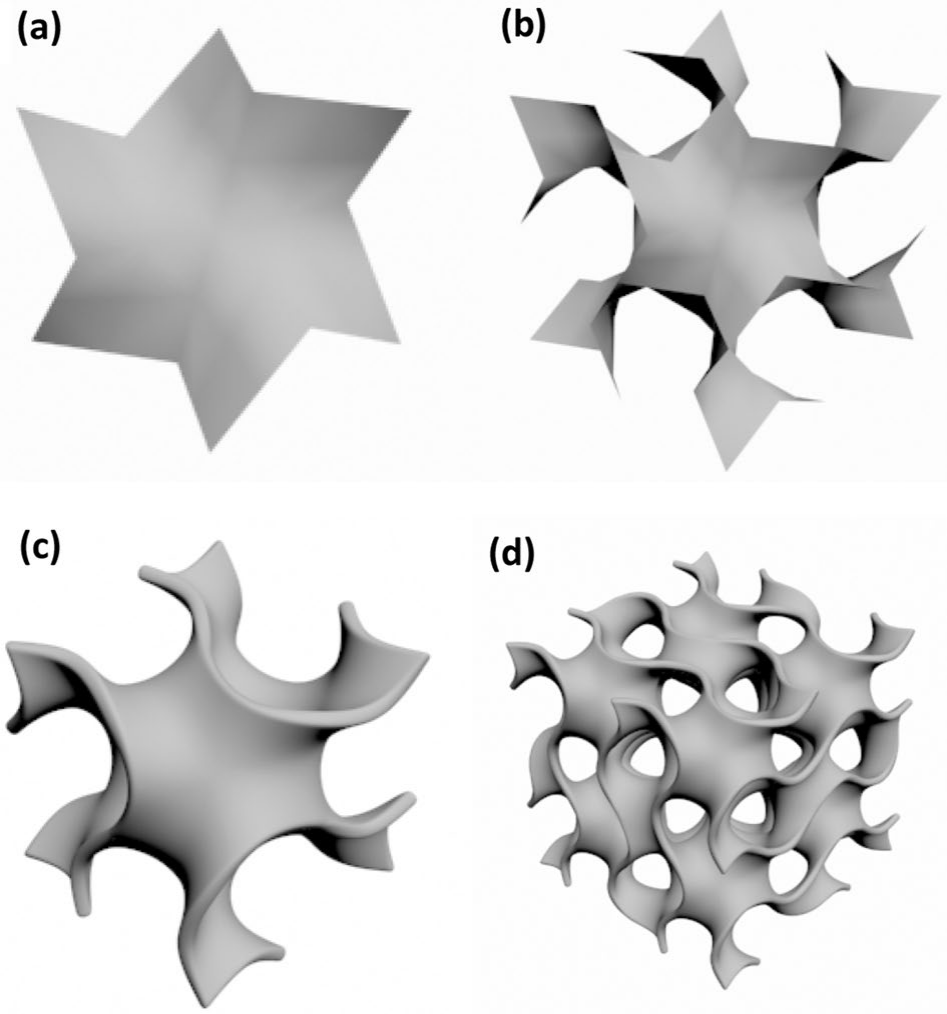
The model of compression sample: (**a**) hexagonal surface; (**b**) hexagonal surface was mirrored, copied, rotated and spliced; (**c**) surface of the unit surface piece was smoothed; (**d**) the element body was arrayed to obtain Gyroid minimal surface element body.

In this paper, 3DMAX software was used to draw G-surface model, and the drawing process was shown in Fig. 8. Firstly, a hexagonal surface was drawn, as shown in Fig. 8a. Secondly, the drawn hexagonal surface was mirrored, copied, rotated and spliced to complete a unit surface piece, as shown in Fig. 8b. Thirdly, the surface of the unit surface piece was smoothed, and the corresponding wall thickness was given to form a G-surface element body, as shown in Fig. 8c. After the completion of Gyroid minimal surface element body, the element body was arrayed to obtain the compressed sample model, as shown in Fig. 8d.

For the compression sample model, the wall thickness was changed to obtain compression samples with various density, so as to analyze the compression performance. The size of compression sample was 15 × 15 × 15 mm. Table 3 shows the parameters of compression model with different wall thicknesses.

**Table 3:** Parameters of compression model with different wall thickness.

Wall thickness *t* (mm)	Density *ρ*	Surface area *C* (mm^2^)
0.1875	0.07495	2683.904
0.375	0.14618	2683.438
0.5625	0.21299	2671.247
0.75	0.27422	2641.798
0.9375	0.33063	2603.874
1.125	0.38208	2556.315

## Results and discussion

**Figure 9:**
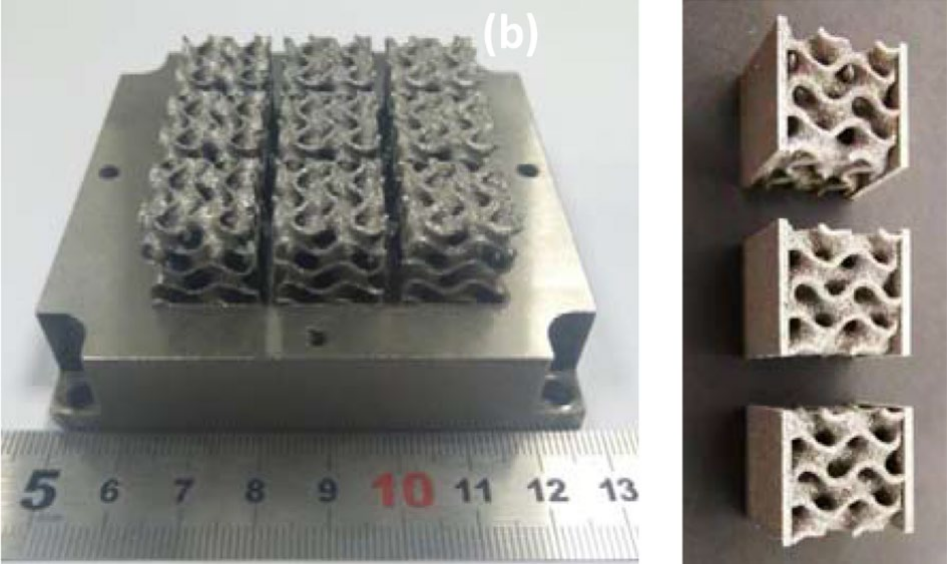
LPBF samples.

Figure 9 shows the printing results. The removed sample was cleaned by ultrasonic cleaning machine, and the industrial alcohol was selected as the cleaning agent. After cleaning for 10 min, the adhesive powder on the sample surface and the stains left during wire cutting were cleaned. The corresponding experiments were carried out on the printed samples.

### Microstructure

**Figure 10:**
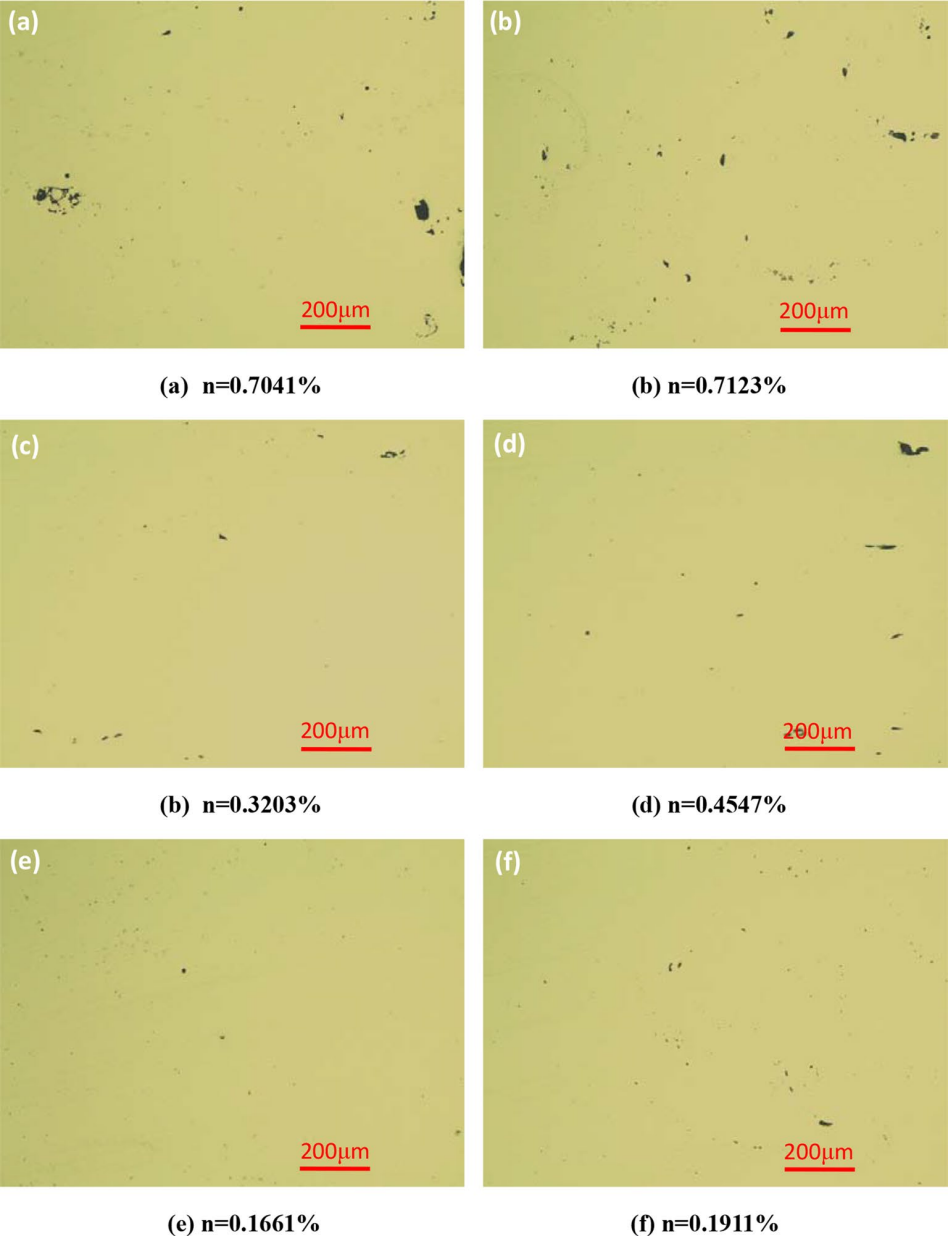
Microstructure of samples under different scanning strategies: (**a**,**b**) Island scanning; (**c**,**d**) helix scanning; (**e**,**f**) slope subarea scanning; (**a**,**c**,**e**) HD; (**b**,**d**,**f**) BD.

In this paper, Zeiss inverted metallographic microscope was used to observe the microstructure of GH3536 nickel base alloy samples under different scanning strategies, as shown in Fig. 10. It can be seen that there are pores in the building direction (BD) and horizontal direction (HD) of the three samples. The main reasons are as follows: (1) the melting speed is too fast, and the liquid metal produces violent thermal expansion, then weld pool flows from the center to the surrounding, finally forming the pore; (2) the local temperature is too high, leading to the gasification of some metal powders^18^; (3) due to the spheroidization, the scanning track is not continuous and causes a large number of pores. Through the analysis of the porosity n of samples by image method, it is found that the density of three types of scanning strategies are above 99%, among which the slope subarea has the highest density, which is above 99.8%; the helix scanning strategy is the second, ranging from 99.5 to 99.7%; while the density of island scanning is relatively worse than that of the first two, which is more than 99.2%.

**Figure 11:**
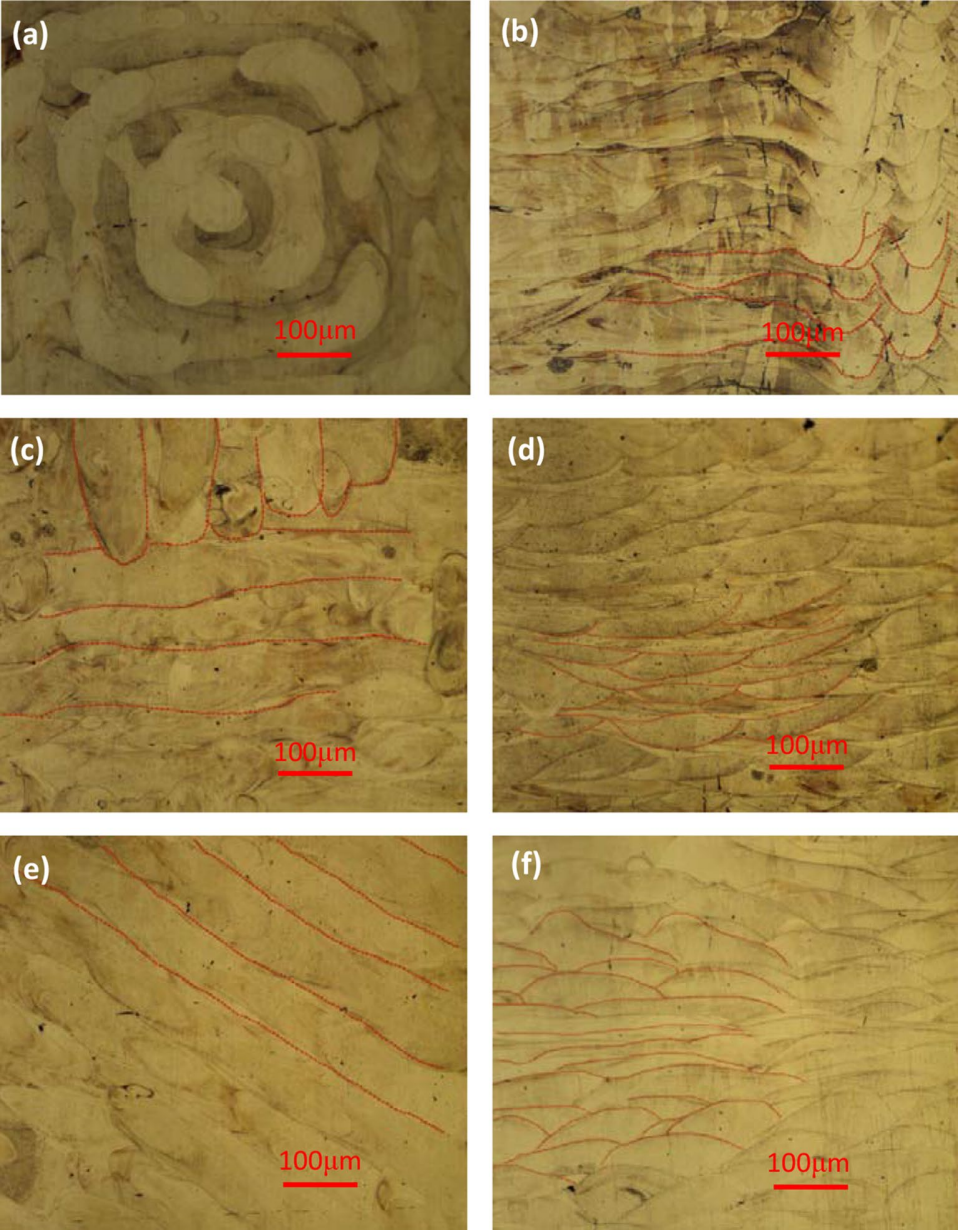
Weld pool of samples under different scanning strategies: (**a**,**b**) helix scanning; (**c**,**d**) Island scanning; (**e**,**f**) slope subarea scanning; (**a**,**c**,**e**) HD; (**b**,**d**,**f**) BD.

The metallographic samples were polished by acid etching solution (HCl + CuCl_2_ + C_2_H_6_O) to obtain the weld pool and organizational structure. Microscope was used to observe the solution pool track morphology of GH3536 nickel base alloy samples under different scanning strategies, as shown in Fig. 11. As shown in Fig. 11a,b, under the helix scanning, morphology of sample presents a recirculated weld pool structure in the HD, which is consistent with the intention of the Fig. 11a. The process of 90° angle of the scanning line is obvious in the BD. Besides, some microcracks appear at the corner because of the high energy concentration and high temperature gradient. As shown in Fig. 11c,d, under the island scanning, there are two vertical scanning directions in the HD; in the BD, weld pool tracks intersect with each other, some of which are vertical to paper and others parallel to paper. This is the characteristics of island scanning and consistent with the Fig. 11c. As shown in Fig. 11e,f, the morphology of the sample under the slope subarea scanning presents a parallel weld pool track in HD. In BD, there is a transformation process of weld pool track from the direction vertical to the paper to that parallel to the paper. Due to the 67° rotation of scanning direction in slope subarea scanning after scanning the current layer, the transformation weld pool is generated in line with Fig. 11e.

**Figure 12:**
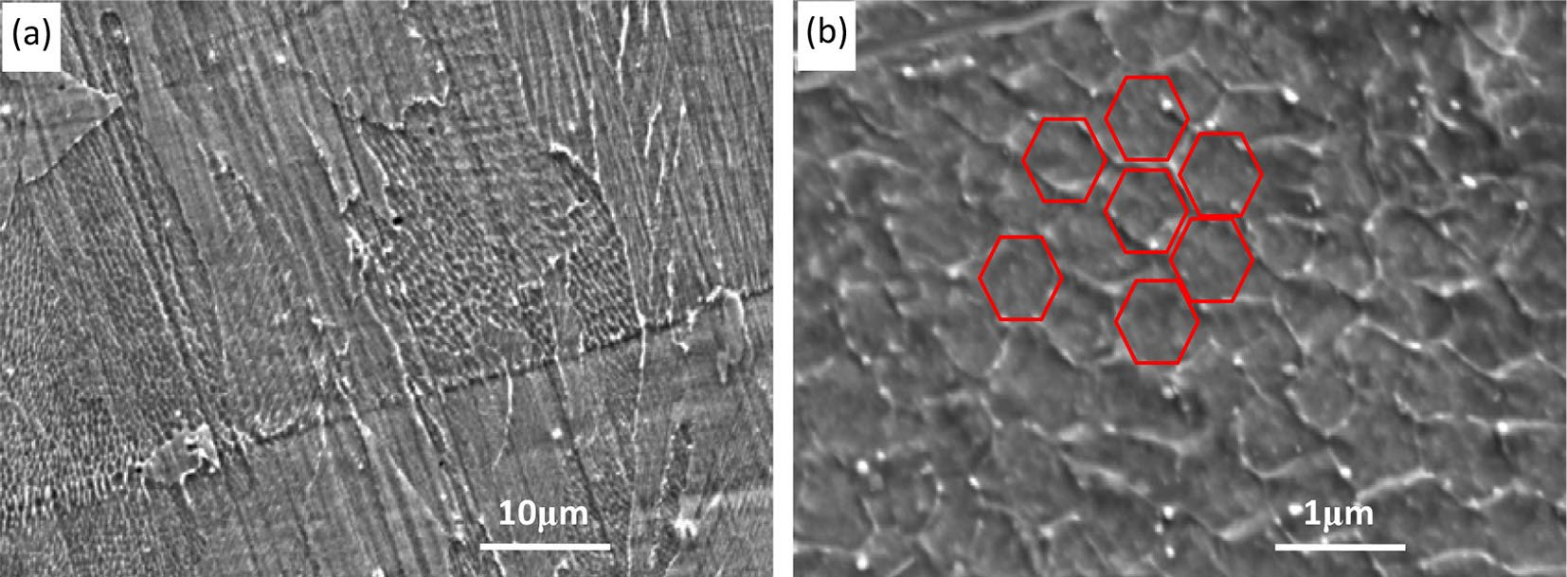
SEM images of typical LPBF GH3536 along BD: (**a**) polycrystalline substructure; (**b**) single crystal substructure.

The microstructure of GH3536 nickel base alloy samples under different scanning strategies was observed, and it is found that different scanning strategies have little effect on microstructure. The organization chart of the sample under the slope subarea scanning was taken for analysis. Figure 12 shows the typical SEM characteristics of LPBF microstructure of GH3536 nickel base alloy in BD. As shown in Fig. 12, the microstructure shows a typical austenite grain-substructure morphology. The austenite grains formed by rapid solidification cross the boundary of adjacent weld pool or channel. For the substructure, the typical honeycomb substructure is evenly distributed in the austenite grains, and the orientation characteristics of the substructure in each austenite crystal are basically the same, while the adjacent austenite intragranular substructure show significant differences in the orientation characteristics. The honeycomb substructure has obvious anisotropy, which is similar to the typical columnar crystal. Its minimum diameter length is about 500 nm- 800 nm, and the longest axis length depends on the size of austenite grain, which often runs through the whole austenite grain.

The formation of this submicron substructure is closely related to the LPBF laser non-equilibrium rapid melting-solidification process: on the one hand, the super fast cooling rate can form fine-grained austenite grains, on the other hand, the heavy metal atoms (such as Mo) in the austenite grains are not homogeneously diffused under the condition of laser rapid cooling. Instead, they cluster at the subgrain boundary interface with relatively high interfacial energy in the microregion, and show the micro morphology similar to the nanoscale sub-grain boundary under the condition of metallographic corrosion^15,19–21^. This kind of sub-micron superfine sub-grain induced by LPBF rapid cooling has an important gain effect on increasing the metal strength. Macroscopically, the sub-grain boundary has higher strength and hardness than the interior of the block; and at room temperature, the more detailed the substructure, the better the reinforcement (while there is a certain viscosity in the grain boundary at high temperature, which is easy to cause the relative sliding of adjacent grains).

**Figure 13:**
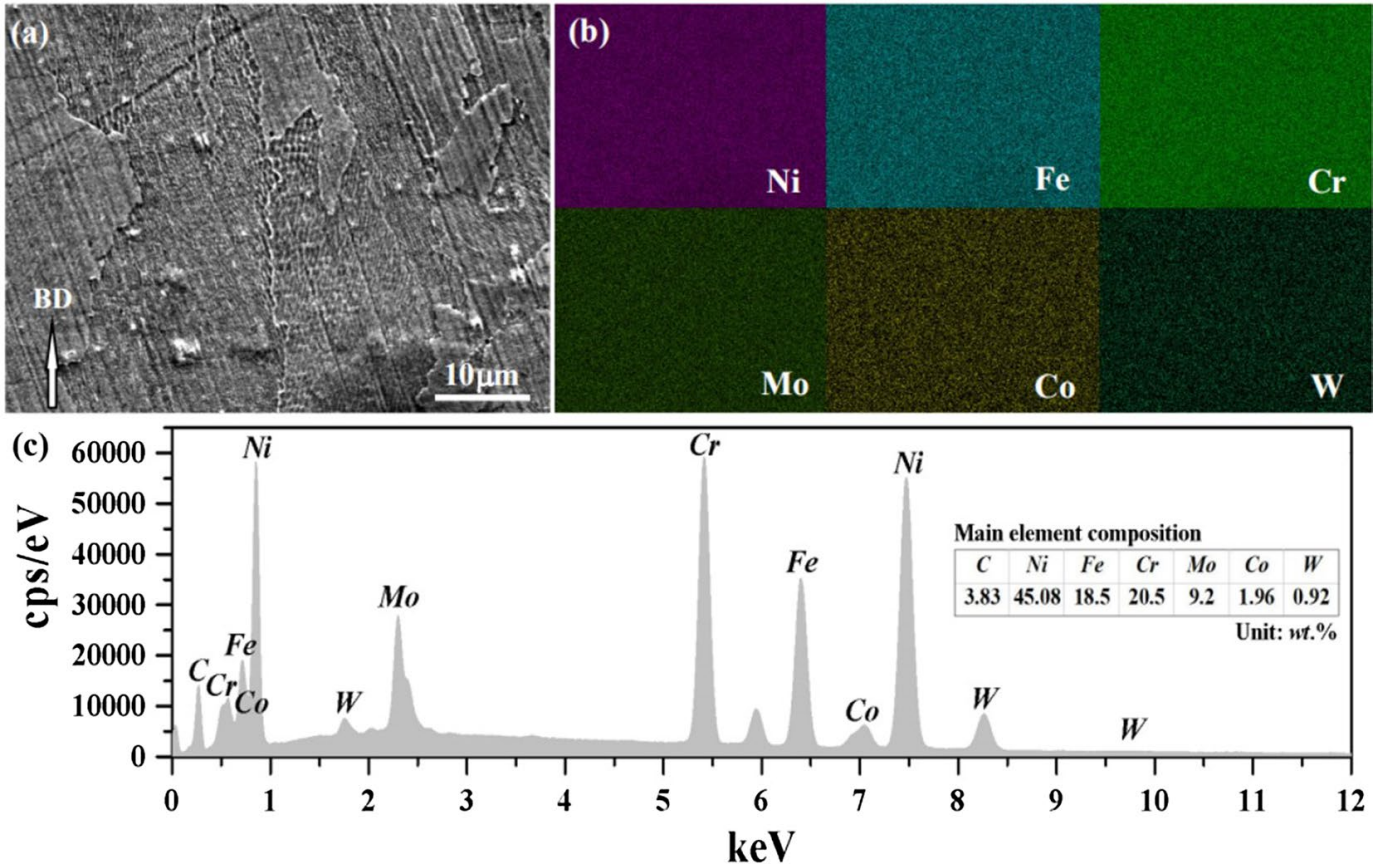
FE-SEM image (**a**) from the cross-section view parallel to BD of the LPBF-built Hastelloy X and its corresponding regional EDS results of Ni, Fe, Cr, Mo, Co, W elemental maps (**b**) and their energy spectrum (**c**).

Figure 13 shows the chemical composition diagram of EDS surface scanning energy spectrum in the LPBF micro region of GH3536 nickel base alloy in the BD. The results show that in the interior and interface region of adjacent austenite grains, there is no significant large-scale component segregation for basic components of GH3536.

### Microhardness

**Figure 14:**
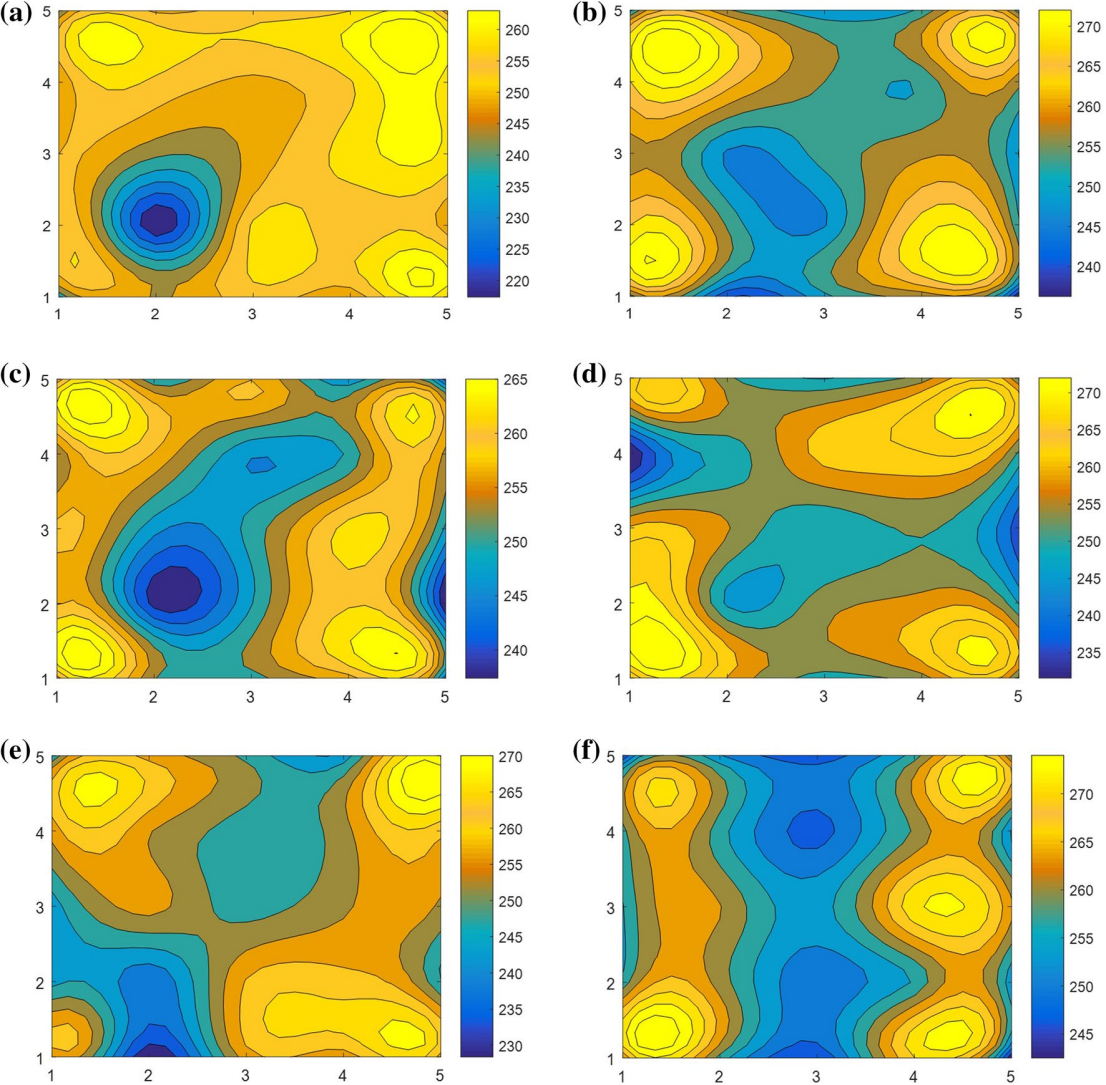
Hardness cloud diagram of samples under different scanning strategies: (**a**,**b**) Island scanning; (**c**,**d**) helix scanning; (**e**,**f**) slope subarea scanning; (**a**,**c**,**e**) HD; (**b**,**d**,**f**) BD.

The hardness cloud diagram of LPBF samples under different scanning strategies is obtained, as shown in Fig. 14.

In this paper, the HXD-1000TMC/LCD micro Vickers hardness tester was used to measure the micro Vickers hardness of GH3536 nickel base alloy samples under different scanning strategies. The load was 300 N and the loading time was 15 s. Each sample was sampled at 25 points. The interval of sampling points was 0.5 mm, forming a 5 × 5 square matrix. Then the average value was calculated. Figure 14 shows the microhardness cloud picture. It can be seen that the overall hardness range of the three scanning strategies is between 245 and 260 HV, with the relatively average hardness distribution. In addition, there are also some points with low values.

**Figure 15:**
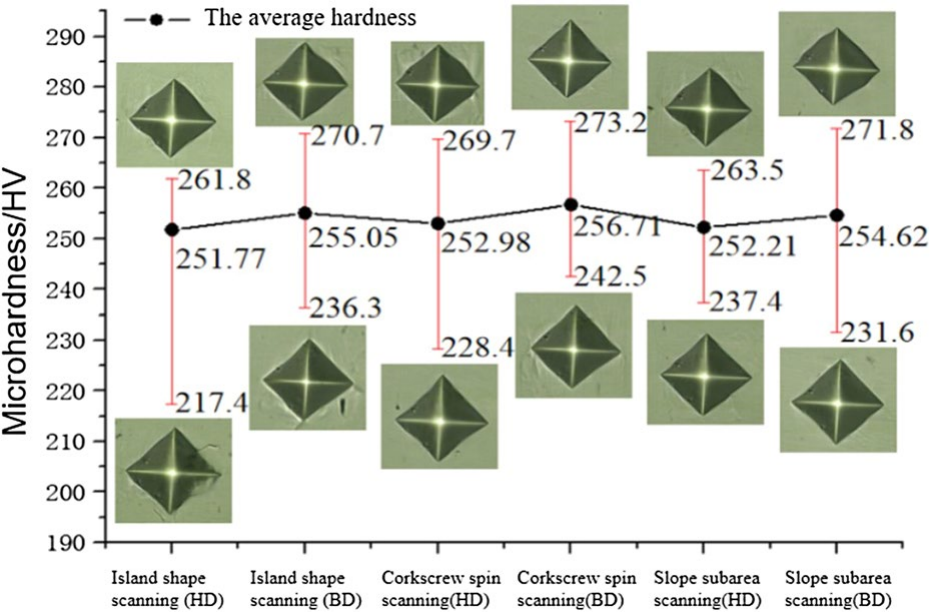
Average hardness curve of samples under different scanning strategies.

The average hardness curves of different scanning strategies are shown in Fig. 15. The average hardness of island scanning, helix scanning and slope subarea scanning are 251.77 HV (HD), 255.05 HV (BD), 252.98 HV (HD), 256.71 HV (BD), 252.21 HV (HD) and 254.62 HV (BD), respectively. The hardness difference between BD and HD surfaces under different scanning strategies is slight, almost no difference. Since micro hardness is the inherent characteristics of materials and closely related to the structure of materials, and the three scanning strategies have no effect on the microstructure, therefore, the microhardness displayed is similar. However, internal pores of the material still affect the hardness of local points. As the microscopic indentation of 217.4 HV in the figure, the indentation edge locally collapses and corresponding cracks appear due to the existence of pores under the indentation position.

### Tensile properties

**Figure 16:**
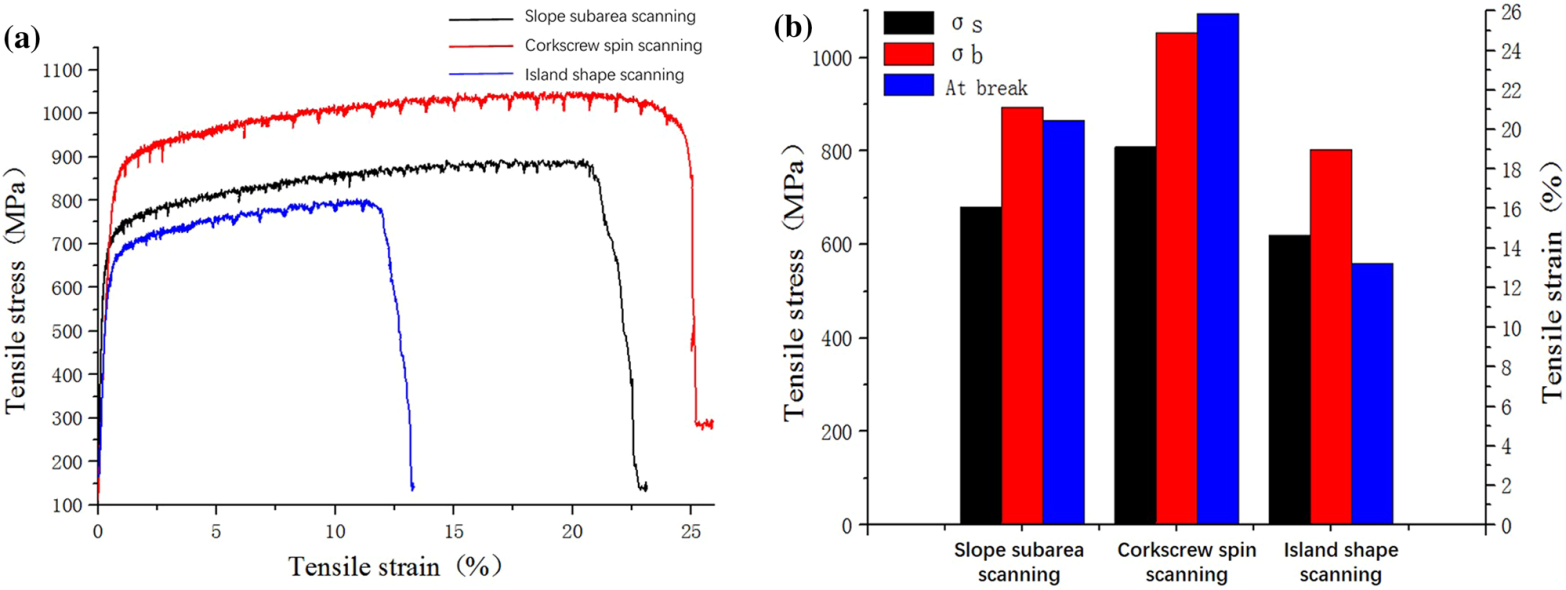
Tensile property of samples under different scanning strategies: (**a**) tensile curve of GH3536 nickel base alloy samples under different scanning strategies; (**b**) histogram of core parameters corresponding to the tensile curve.

Tensile properties of GH3536 nickel base alloy samples under different scanning strategies were measured as shown in Table 4 and Fig. 16. Figure 16a shows the tensile curve of GH3536 nickel base alloy samples under different scanning strategies, and Fig. 16b shows the histogram of core parameters corresponding to the tensile curve. Table 5 lists the detailed parameter information of the tensile sample under different scanning strategies. It can be seen that the tensile limit, yield limit and maximum strain of tensile sample under the helix scanning are 1052.5 MPa, 808.8 MPa and 23.1% respectively, which are much higher than those obtained by slope subarea scanning and island scanning. Among the three scanning strategies, the performance of the sample under the helix scanning is the best, followed by slope subarea scanning, and the performance of sample under the island scanning is the worst. The reason is as follows: under the helix scanning, the stretching direction is the same as that of the weld pool track, and the stretching area is composed of continuous weld pool tracks in the same direction, so the stretching performance is the best. In the island scanning, the scanning area is divided into several small square sub areas, and there are many sub area weld junctions in the stretching area. In other words, there are weld pool tracks parallel to the stretching direction and weld pool tracks perpendicular to the stretching direction, which are connected with each other. During the stretching, the mechanical properties of the junction and weld pool tracks perpendicular to the stretching direction are prone to fracture, resulting in the worst tensile performance. However, there is the 67° difference between the track layers of slope subarea scanning, so there is a continuous weld pool track parallel and perpendicular to the stretching direction, and the track direction is changed constantly and alternately. Therefore, tensile properties of slope subarea scanning are between helix scanning and island scanning.

**Table 4:** Tensile parameter of samples under different scanning strategies.

	Tensile strain at break (%)	Tensile strain at maximum load (%)	Modulus (automatic Young's) (GPa)	Tensile stress at yield (offset 0.2%) (MPa)	Tensile stress at maximum load (MPa)
Slope subarea	23.07291 ± 1.5	20.45168 ± 1.8	234.88362 ± 12.5	680.31890 ± 15.8	894.39191 ± 20.8
Helix	25.88664 ± 1.8	17.59046 ± 1.2	150.44490 ± 10.7	808.83446 ± 20.8	1053.51660 ± 25.6
Island	13.23293 ± 1.2	10.91204 ± 1.6	161.17493 ± 10.4	619.75072 ± 15.3	802.98224 ± 20.4

**Table 5:** Elastic modulus of Gyroid structures with different wall thickness.

	Wall thickness t/mm					
Elastic modulus/GPa	0.1875	0.375	0.5625	0.75	0.9375	1.125
**Compression direction**						
BD	0.057	0.111	0.134	0.181	0.212	0.255
HD	0.052	0.092	0.129	0.166	0.194	0.203

### Solid compression performance

**Figure 17:**
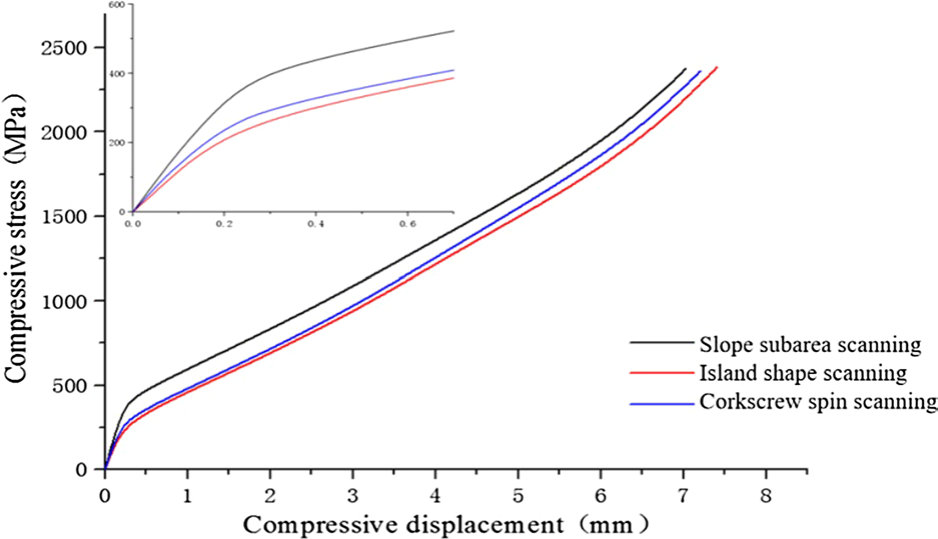
Compression curve of samples under different scanning strategies.

The quasi-static compression properties of GH3536 nickel base alloy samples under different scanning strategies were measured. The stress-displacement curve is obtained, as shown in Fig. 17. It can be seen that the overall trend of the compression performance of the samples under different scanning strategies is relatively consistent. There are obvious elastic stages in the three compression curves, and the intermediate plastic stage is relatively long due to the excellent plasticity of GH3536. But the elastic modulus in the early elastic stage is different. The elastic modulus E of samples under three scanning strategies is 1.68 GPa (slope subarea), 1.38 GPa (Helix) and 1.19 GPa (Island), respectively. The elastic modulus of the sample under the slope subarea scanning is the largest, while the elastic modulus of the sample under the island scanning is the smallest. The compression direction of the three is all in BD, so the weld pool formed by scanning strategy has little influence on the compression performance. The defects such as pores produced by scanning strategy lead to different elastic modulus. Compared with the previous density, it can be found that the higher the density, the greater the elastic modulus of compression.

### Formability and compressibility of Gyroid minimal surface

By comparing the effects of three scanning strategies on the structure and properties of the parts, the results show that the density of the parts obtained by slope subarea scanning is higher than that of the other two strategies, while the microstructure and microhardness are similar under the three scanning strategies; the yield strength and tensile strength of the parts under the slope subarea scanning are higher than that of parts under the island scanning strategy and lower than that of parts under the helix scanning strategy in the tensile performance; in the solid compression performance, the elastic modulus and compressive strength under the slope subarea scanning are higher than those under other two strategies. Therefore, for the sake of comprehensive consideration, slope subarea scanning is selected as the best strategy when preparing LPBF additive to manufacture GH3536 structural parts.

The complex structures of Gyroid minimal surfaces with different wall thicknesses were fabricated by 3D printing with slope subarea scanning, and the quasi-static compression experiments of Gyroid minimal surfaces with different wall thicknesses were carried out in the BD and HD.

**Figure 18:**
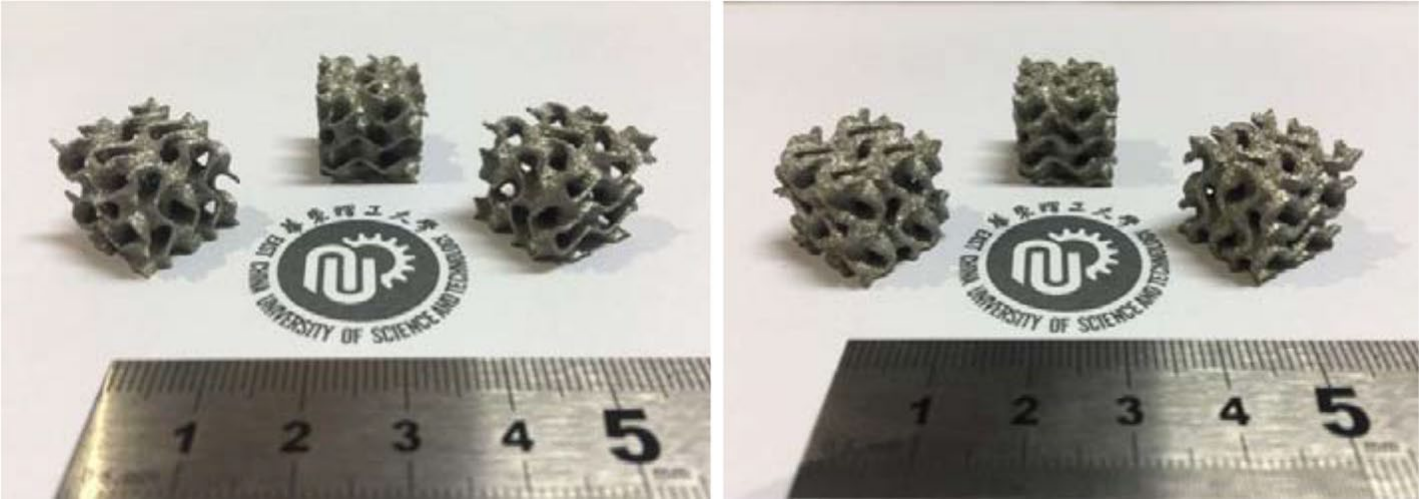
Formed samples of Gyoid complex surface.

As shown in Fig. 18, six Gyroid minimal surfaces with different wall thicknesses are manufactured by LPBF. They have complete structure, and the surface with the thinnest wall thickness t = 0.1875 mm has the excellent forming quality. Therefore, the self-designed LPBF equipment in this paper has good formability and can manufacture thin-walled structure and functional parts with complex curved surface structure.

**Figure 19:**
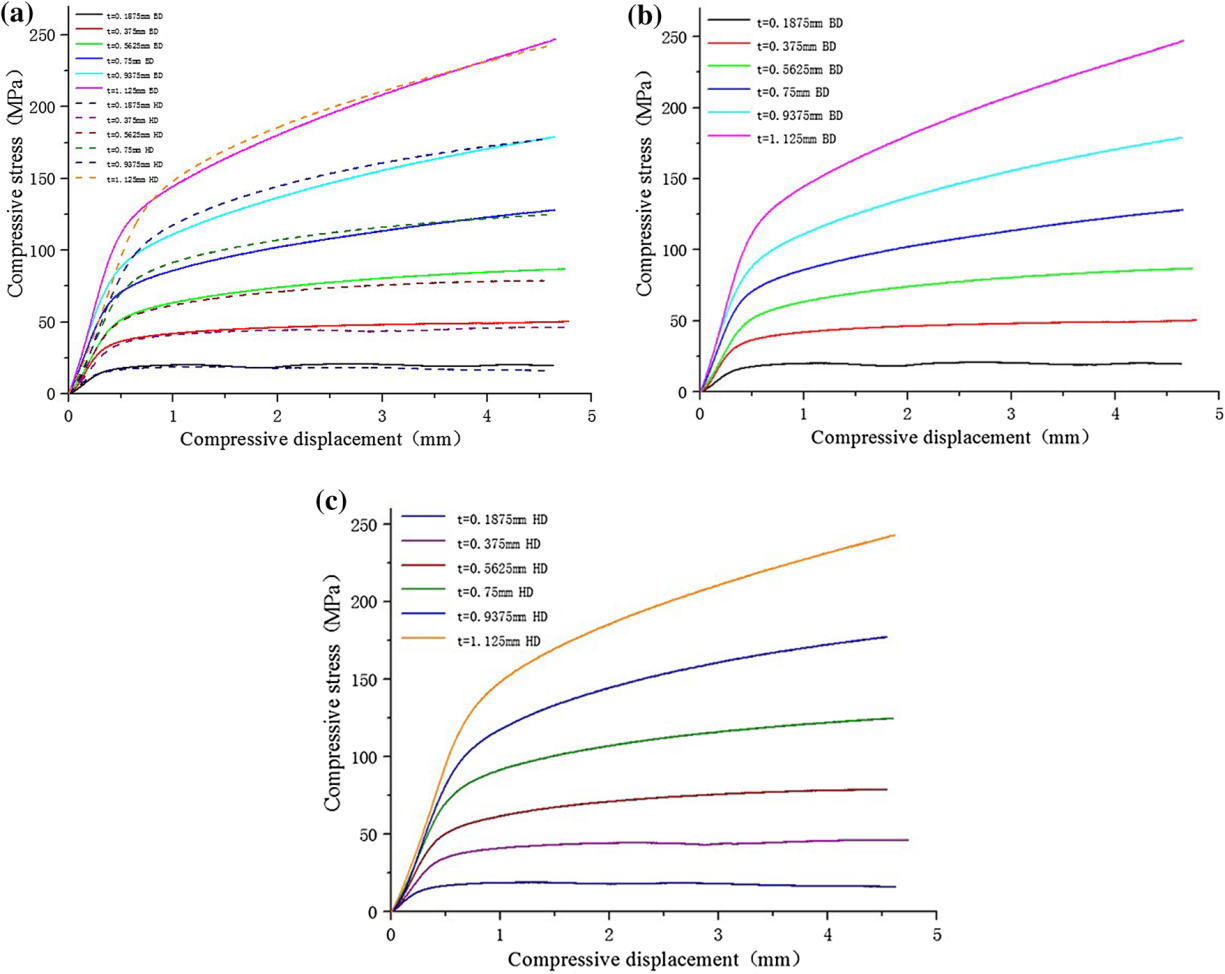
Compression curves of Gyroid surfaces with different wall thickness: (**a**) the compression strength curve of solid structure; (**b**) the compression strength curve of G-surface structure in BD direction; (**c**) the compression strength curve of G-surface structure in HD direction.

Through the quasi-static compression experiment of Gyroid minimal curved surface structure with different wall thickness, the stress displacement compression performance curve is shown in Fig. 19.

**Figure 20:**
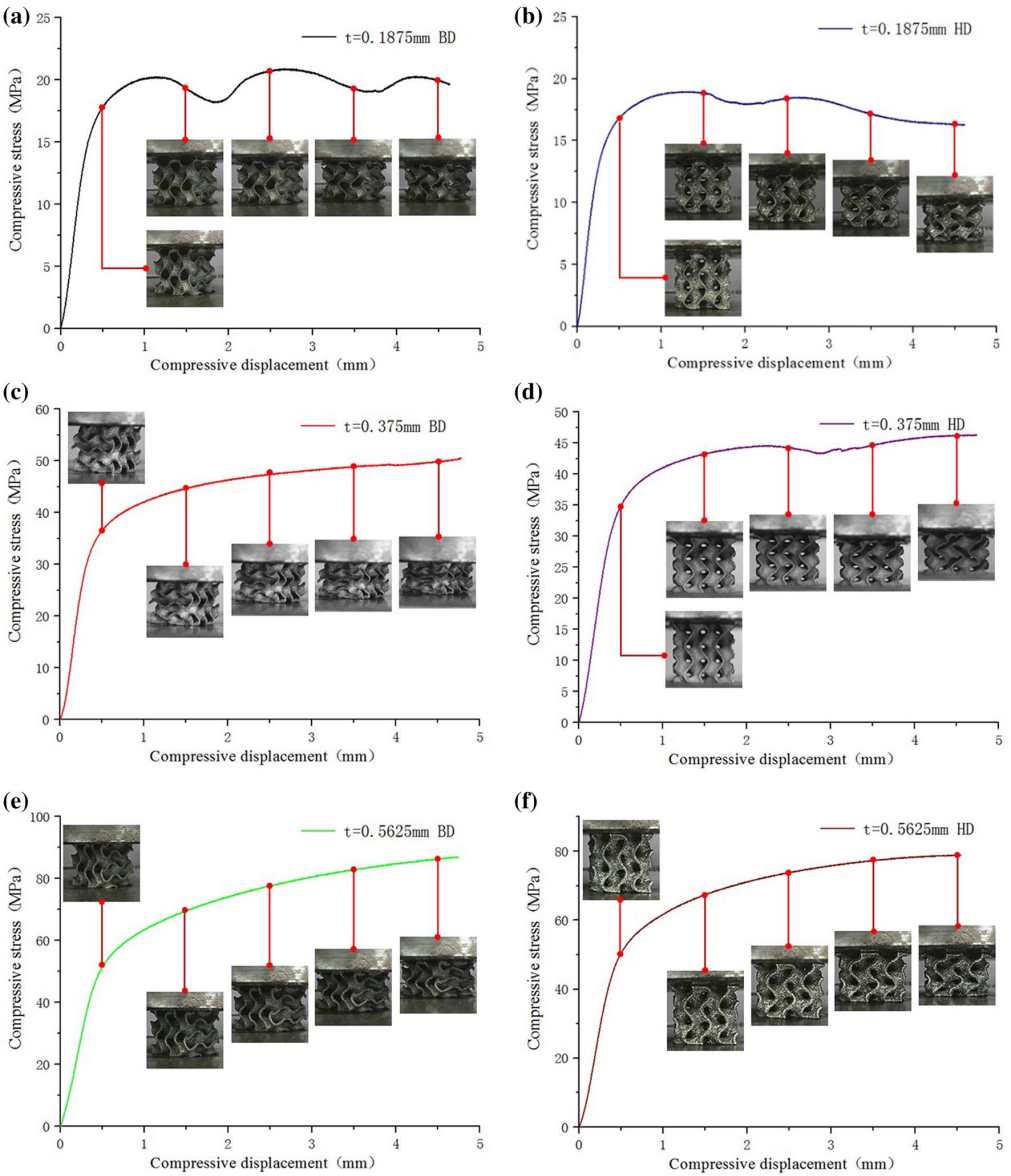

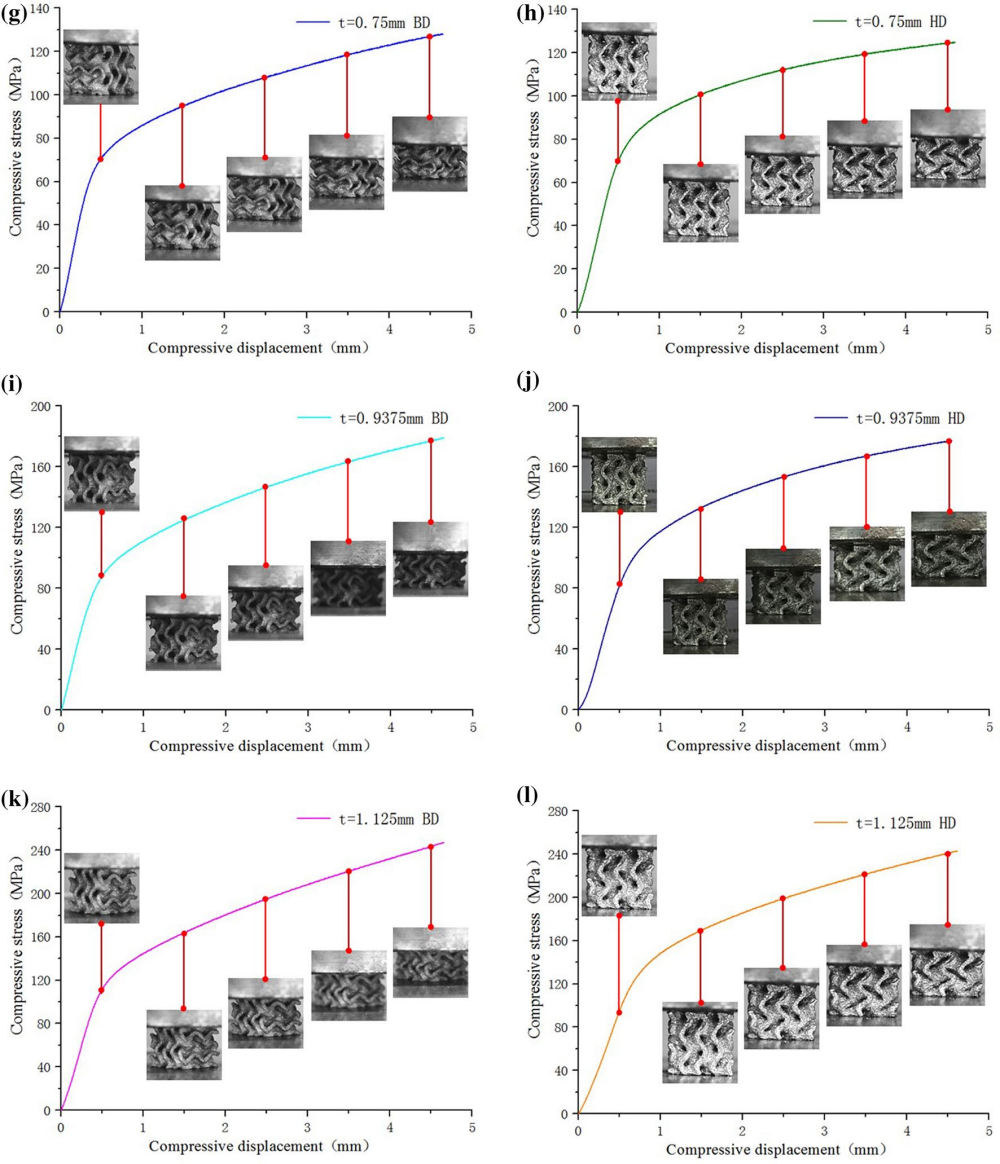
Compression deformation of Gyroid surfaces with different wall thickness: (**a**) t = 0.1875 mm BD; (**b**) t = 0.1875 mm HD; (**c**) t = 0.375 mm BD; (**d**) t = 0.375 mm HD; (**e**) t = 0.5625 mm BD; (**f**) t = 0.5625 mm HD; (**g**) t = 0.75 mm BD; (**h**) t = 0.75 mm HD; (i) t = 0.9375 mm BD; (**j**) t = 0.9375 mm HD; (**k**) t = 1.125 mm BD; (**l**) t = 1.125 mm HD.

It can be seen from Fig. 19a: compared with the compression curve of solid structure, the elastic modulus of G-surface structure in the elastic stage is less than that of solid structure, and the rising trend of G-surface structure in the intermediate plastic stage is far less than that of solid structure, and the compression strength is also less than that of solid structure. However, the smooth plastic stage of the compression curve of G-surface structure shows good energy absorption characteristics. As the wall thickness increases (the relative density decreases), the mechanical properties of the Gyroid structure also increase, as shown in Fig. 19b,c. The elastic modulus of the structure with different wall thickness is obtained by processing (a)–(l) curves in Fig. 20, as shown in Table 5.

As shown in Fig. 20, in BD, the elastic modulus of Gyroid structure increases gradually with the increase of wall thickness (the decrease of relative density); in HD, the elastic modulus of G-surface structure also increases gradually with the increase of wall thickness (the decrease of relative density). However, at the same wall thickness, the compressive modulus in BD is slightly higher than that in HD, and the compressive strength in BD is also slightly higher than that in HD. This is because BD is the main direction of the grain growth orientation produced by LPBF, that is to say, under the guidance of the thermal processing temperature gradient, grain grows towards the direction of the upward layering of the weld pool. Therefore, the mechanical properties in the grain growth direction (mainly reflected by a large number of columnar crystals in this direction) are higher than those in the vertical grain growth direction.

It should be noted that for the G-surface structure with thin wall thickness, as shown in Figs. 19b and 20a, the stress-displacement stage of the sample under compression shows multiple "cyclic instability" characteristics. The occurrence of this intermittent instability or relaxation phenomenon has obvious periodic characteristics. Combined with the deformation observation of samples with different compression displacement, it is considered that when the wall thickness is thinner, G-surface structure has a periodic evolution of "elastic deformation—plastic instability—fracture failure—plastic deformation—deformation strengthening" layer by layer during the compression process. For the thicker wall thickness of the G-surface structure, there is no layered fracture, but an evolution process of "elastic deformation- plastic deformation- deformation strengthening" layer by layer. Therefore, the compression curve of G-surface structure with the thin-walled shows a wavy shape.

**Figure 21:**
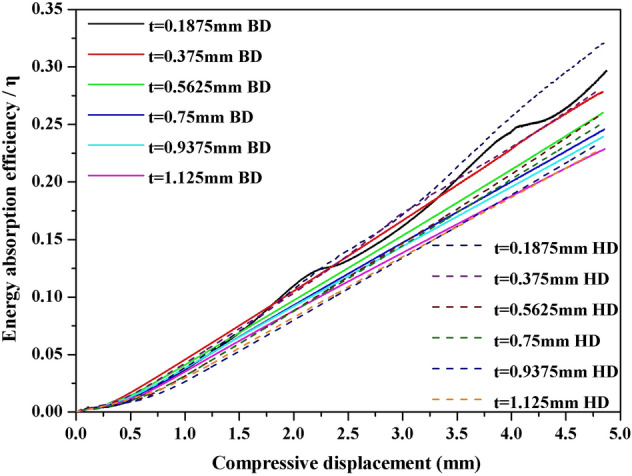
Energy absorption efficiency curve of Gyroid surface with different wall thickness.

The smooth and long-term plastic stage of the static-quasi compression curve of G-surface shows good energy absorption characteristics. In the compression of the external load, the flow stress level is low due to large deformation, and the impact energy is transformed into the energy such as deformation and cracking of holes in G-surface structure. Therefore, G-surface structure is a good energy absorption structure. To clearly observe the energy absorption characteristics of different wall thickness, the energy absorption efficiency *η* is used as the evaluation method: the function of energy absorption capacity *E* is shown in Eq. (2), and the function of energy absorption efficiency *η* is shown in Eq. (3).

2 $$ E = \int\limits_{0}^{{\varepsilon_{{\text{m}}} }} {\sigma d\varepsilon } $$

3 $$ \eta = \frac{E}{{\sigma_{{\text{m}}} }} = \frac{1}{{\sigma_{{\text{m}}} }}\int\limits_{0}^{{\varepsilon_{{\text{m}}} }} {\sigma d\varepsilon } $$

where *ε*_m_ is the strain at a certain time; *σ*_m_ is the corresponding stress at that time; *ε* and *σ* are compression stress and compression strain respectively;

Through the numerical calculation of the curve by Eq. (3), the energy absorption efficiency curve is obtained, as shown in Fig. 21. With the increase of strain, the energy absorption efficiency of the G-surface structure increases. When the wall thickness decreases, the energy absorption efficiency of the G-surface structure increases. That is to say, the more gentle the compression curve, the higher the energy absorption efficiency, and the better the energy absorption characteristics.

## Conclusion

The process of GH3536 nickel base alloy was studied by self-designed LPBF equipment, and the effects of different scanning strategies on the microstructure, porosity and properties of GH3536 were explored.

1. The results show that the density of the parts obtained by slope subarea scanning is higher than that of parts by the other two strategies, and the microstructure of the parts by slope subarea scanning is similar to that of parts by the other scanning strategies;
2. In the aspect of tensile properties, the yield strength and tensile strength under slope subarea scanning are higher than that under island scanning, and lower than that under helix scanning;
3. The elastic modulus and compressive strength of the parts by slope subarea scanning the are higher than those of parts under the other two strategies. Therefore, slope subarea scanning is the optimal strategy.

The model experiment of G-surface structure was carried out by slope subarea scanning. Six G-surfaces with different wall thicknesses were manufactured in the experiment. Both of them had the complete structure and the good quality. Then, the compression performance of G-surface structure was studied, and the results show that:

1. Compared with the compression curve of solid structure, the compression performance of G-surface structure is smaller than that of solid structure, the compression strength of G curved can only reach about 20% of solid structure, but the smooth compression curve of G-surface structure shows good energy absorption characteristics;
2. With the increase of the wall thickness (the decrease of the relative density), the mechanical properties of the G-surface structure also increase, while the energy absorption efficiency decreases;
3. At the same wall thickness, the compressive modulus in BD is higher than that in HD, and the compressive strength in BD is slightly higher than that in HD.
